# Early and Progressive Spinal Cord Atrophy in Spinocerebellar Ataxia Type 1

**DOI:** 10.1002/mds.70294

**Published:** 2026-04-09

**Authors:** Colette J.M. Reniers, Teije H. van Prooije, Kirsten C.J. Kapteijns, Jack J.A. van Asten, Caterina Mariotti, Lidia Sarro, Anna Nigri, Marina Grisoli, Giulia Coarelli, Alexandra Durr, Tom Scheenen, Bart van de Warrenburg

**Affiliations:** ^1^ Department of Neurology, Donders Institute for Brain, Cognition, and Behavior Radboud University Medical Center Nijmegen The Netherlands; ^2^ Department of Medical Imaging Radboud University Medical Center Nijmegen The Netherlands; ^3^ Neurology Department Fondazione IRCCS Istituto Neurologico Carlo Besta Milan Italy; ^4^ Neuroradiology Unit Fondazione IRCCS Istituto Neurologico Carlo Besta Milan Italy; ^5^ Sorbonne Université, Paris Brain Institute ‐ ICM, Inserm, CNRS, APHP Hopital de la Pitié‐Salpêtrière Paris France; ^6^ Department of Genetics Hopital de la Pitié‐Salpêtrière, APHP Paris France

**Keywords:** early biomarker, neurodegeneration, spinal cord, spinocerebellar ataxia type 1

## Abstract

**Background:**

Spinocerebellar ataxia type 1 (SCA1) is a rare, autosomal dominant neurodegenerative disorder characterized by progressive cerebellar and brainstem degeneration. Previous studies have shown that spinal cord atrophy is also a key aspect of SCA1 neuropathology. Magnetic resonance imaging (MRI)‐based spinal cord measurements could, therefore, serve as staging or monitoring biomarkers. However, previous findings were limited to cross‐sectional analyses, and longitudinal changes remain unexplored.

**Objectives:**

This study investigates both cross‐sectional and longitudinal cervical spinal cord alterations in SCA1 mutation carriers compared to healthy controls, evaluating the utility of this biomarker.

**Methods:**

Baseline and 1‐year MRI changes were assessed in 40 controls, 16 preataxic and 58 symptomatic SCA1 mutation carriers. T1‐weighed images were processed with FreeSurfer and the Spinal Cord Toolbox. We related Z‐scores to disease duration, calculated standardized response means, and analyzed clinico‐genetic associations using a linear mixed model.

**Results:**

The three groups differed significantly in cross‐sectional area (CSA) at all levels at baseline. Over time, CSA at levels C1 and C2 decreased in the preataxic and symptomatic groups compared to controls. Preataxic carriers already showed pronounced spinal cord atrophy, whereas pontine changes emerged later in the disease course. Standardized response means were highest for CSA at C2 in preataxic stage, whereas this was pontine volume at symptomatic stage, indicating region‐specific biomarkers across disease stages.

**Conclusion:**

Cervical spinal cord atrophy is an early and progressive feature of SCA1, detectable before clinical onset, providing a promising imaging biomarker for early disease stages. Our findings suggest a caudal to rostral progression of atrophy in SCA1. © 2026 The Author(s). *Movement Disorders* published by Wiley Periodicals LLC on behalf of International Parkinson and Movement Disorder Society.

Spinocerebellar ataxia type 1 (SCA1) is a rare, autosomal dominant, neurodegenerative disorder within the larger group of spinocerebellar ataxias.^1^ These disorders are primarily characterized by progressive motor impairment, including gait ataxia, dysarthria, and impaired coordination. Among the different SCA subtypes, SCA1 has the most rapidly progressive disease course, with clinical disease progression measurable within 1 year.^2^, ^3^ Currently, there is no disease‐modifying treatment available for SCA1. Symptomatic and quality‐of‐life oriented interventions, such as rehabilitation tools, are being explored to improve functional independence and well‐being.^4^ In parallel, several promising genetic therapies are under investigation, including antisense oligonucleotide (ASO) strategies aiming at reducing mutant protein levels.^5^ In anticipation of these disease‐modifying clinical trials, the identification of objective and sensitive biomarkers to monitor disease progression and treatment efficacy across the different disease stages is of critical importance.

Although much research has focused on cerebellar and brainstem degeneration in SCAs, there is growing recognition of the involvement of the spinal cord in these disorders.^6^, ^7^, ^8^ Postmortem studies have revealed that spinal cord pathology is a consistent feature in patients with SCA1.^9^ Spinal cord atrophy is because of degeneration of anterior horn cells, dorsal column pathways, and corticospinal tracts, reflected by clinical features such as deep sensory disturbances, muscle atrophy, fasciculations, and spasticity.^10^, ^11^ Magnetic resonance imaging (MRI) studies have corroborated these pathological findings, demonstrating spinal cord atrophy in patients with SCA1. For example, previous cross‐sectional studies have shown a significant reduction in spinal cord cross‐sectional area (CSA) at the cervical level, mainly at C2, in SCA1 patients compared to healthy controls.^6^, ^7^ In addition to CSA, spinal cord eccentricity provide complementary information on spinal cord shape and is considered a surrogate marker of dorsal column involvement. This is relevant as involvement of sensory pathways has been reported in SCA1.^12^, ^13^

In SCA3, recent studies suggested that the pathological process in this disease might in fact first emerge in the spinal cord and lower brainstem regions before ascending to upper brainstem and the cerebellum,^8^, ^14^ a scenario that might be possible in SCA1 as well. This also suggests that spinal cord volumetry could serve as a potential early biomarker of disease progression. However, to date, no longitudinal imaging studies have examined spinal cord changes over time in this population.

This study aims to address this gap by investigating both cross‐sectional and longitudinal changes in the spinal cord in preclinical and symptomatic individuals with SCA1 compared to healthy controls. Specifically, we focus on spinal cord CSA and eccentricity at multiple cervical levels to determine whether spinal cord imaging could serve as a robust biomarker for disease stage and progression. By tracking spinal cord changes over time and comparing these changes to brainstem and cerebellar measures, we aimed to explore its potential as an objective and quantifiable marker for future therapeutic trials, and to gain more insight into atrophy progression in SCA1.

## Methods

### Participants

For this study, data from three previously acquired natural history cohorts, from the Radboudumc in Nijmegen,^3^ the Fondazione IRCCS Istituto Neurologico Carlo Besta in Milan,^15^ and the Paris Brain Institute^16^ were pooled to investigate spinal cord differences between individuals carrying an *ATXN1* repeat expansion and healthy controls. Inclusion criteria were 18 years or older, a confirmed molecular genetic diagnosis of SCA1, and absence of other neurological conditions. Both symptomatic and preataxic individuals were included. The number of CAG repeats in the *ATXN1* gene was determined for all participants. Data from baseline and 1‐year follow‐up assessments were included in the present analysis.

### Clinical Characteristics and Assessments

Age at disease onset and disease duration were based on participants' self‐reported first symptoms. For individuals without self‐reported gait or balance disturbances but with clinical signs of ataxia, disease duration was set to 0 years. In preataxic individuals, defined as SCA1 mutation carriers with a Scale for the Assessment and Rating of Ataxia (SARA) score below 3,^17^ estimated age at onset was calculated using a previously reported formula.^18^ The SARA was administered both at baseline and 1‐year follow‐up to evaluate the presence and severity of ataxia.^19^ This clinical scale has a maximum score of 40, with higher scores indicating more severe ataxia. Non‐ataxic features were evaluated using the Inventory of Non‐Ataxia Signs (INAS).^20^

### MRI Acquisition

We used available baseline and 1‐year follow‐up T1‐weighted images (T1w) acquired using different 3 T MRI systems across the three sites. In Nijmegen, structural imaging included a high‐resolution three dimensional (3D) T1‐weighted magnetization‐prepared rapid gradient‐echo (MPRAGE) pulse sequence (pulse repetition time [TR] = 2300 ms, echo time [TE] = 4.48 ms, inversion time [TI] = 950 ms, flip angle = 12º, voxel size = 0.9 mm isotropic, field of view [FOV] = 256 × 256 mm^2^, acquisition orientation = sagittal, slices = 192, acquisition orientation = sagittal), acquired on a Magnetom Prisma‐fit (Siemens Healthineers, Erlangen, Germany). In Paris, a similar 3D MPRAGE sequence was used (TR = 2530 ms, TE = 3.65 ms, TI = 900 ms, flip angle = 9º, voxel size = 1 mm isotropic, FOV = 256 × 256 mm^2^, acquisition orientation = axial, slices = 160), acquired on a Magnetom Trio (Siemens Healthineers, Erlangen, Germany) using a standard 32‐channel receive headcoil. In Milan, a high‐resolution 3D T1‐weighted gradient‐echo image was acquired on a Achieva 3T clinical scanner (Philips Healthcare, Best, The Netherlands) (TR = 9.8 ms, TE = 4.6 ms, flip angle = 8º, voxel size = 1 mm isotropic, FOV = 240 × 240 mm^2^, acquisition orientation = sagittal, slices = 185). Scans were aligned to the brain, placing the spinal cord slightly off isocenter, however, spatial offsets of C1–C4 were small and unlikely to cause relevant geometric distortion after standard system corrections. In axial 3D acquisitions, C1–C4 vertebral levels were covered completely in 92.9% of participants, whereas sagittal acquisitions covered C1–C4 in 75% (Milan) and 79.6% (Nijmegen).

### Spinal Cord Morphometry

Brain T1w images were manually checked for artifacts or motion‐related distortions; five scans were excluded (1 control/4 symptomatic). Spinal cord segmentation and morphometry were performed with the Spinal Cord Toolbox,^21^ using the DeepSeg algorithm (Fig. S1),^22^ a specialized model created with deep learning frameworks using a contrast‐agnostic segmentation. The segmentation was checked manually with the integrated quality control tool and corrected whenever necessary with FSLeyes (FSL, version 6.0.7.017).^23^, ^24^ Vertebral levels were labelled via spinal cord straightening, C2–C3 disc detection, and PAM50 template registration.^25^, ^26^ Finally, the spinal cord was un‐straightened and the labels were saved. The mean angle‐corrected CSA and eccentricity were computed per cervical vertebral level.^27^ The vertebral level that was only partially captured within the FOV, the most inferior vertebra, was excluded from further analysis. Longitudinal values were evaluated per vertebral level and group using a ± 2 standard deviation (SD) criterion based on the group‐specific mean change between baseline and 1‐year follow‐up. If a level exceeded this threshold, that level and all inferior levels for that subject were excluded, resulting in the exclusion of 23 CSA (10 unique participants) and 10 eccentricity (6 unique participants) measurements. Usable levels varied slightly per participant because of differences in image coverage (Table S1).

### Brain Morphometry

For brain morphometry, FreeSurfer version 7.0^28^ was used to estimate volumes of regions of interest, including the brainstem, cerebellar white matter, cerebellar cortex, medulla oblongata, pons, and superior cerebellar peduncles (SCP). The standard recon‐all pipeline was used, supplemented by the brainstem module to ensure accurate segmentation of infratentorial structures.^29^ All datasets were reprocessed to ensure consistency of segmentation across participants and visits. We used the brainstem structures, which are hereafter referred to as “brainstem structures.” For cerebellar sub‐segmentation, CerebNet, a deep‐learning–based algorithm, was used to segment the cerebellum into cerebellar cortex and cerebellar white matter.^30^ All volumetric magnetic resonance (MR) measures were normalized for estimated intracranial volume.

### Statistical Analysis

In our primary analyses we included (1) group‐level comparisons across ataxic, preataxic, and healthy controls; (2) longitudinal within‐group progression and sensitivity‐to‐change (standardized response mean [SRM]) over 1 year; and (3) age‐adjusted comparisons across vertebral levels and brainstem/cerebellar structures. Secondary analyses examined structure‐clinical associations, and clinically relevant subgroups, as well as sample size/power simulations for 1‐year trial scenarios.

Baseline spinal cord CSA and eccentricity were compared between SCA1 participants and controls using a linear mixed‐effects models (*lmerTes*t)^31^ with each cervical segment as dependent variable, group (control, preataxic, and symptomatic), age and sex as fixed effects, and site as a random intercept. Significant group effects were followed by Holm‐corrected pairwise comparisons using estimated marginal means. Confidence intervals (CI) for fixed effects and contrasts were calculated using the Wald method.^32^, ^33^

To assess longitudinal changes of spinal cord CSA and eccentricity, separate Linear Mixed Models were fitted for each cervical segment to evaluate within‐group progression, with time, age and sex as fixed effects, and subject and site as random intercepts to account for repeated measures. Significant group × time interactions were followed by Holm‐corrected post‐hoc tests.

To explore the temporal dynamics of regional CSA and brainstem structure, while taking into account age‐related effects, all metrics were z‐transformed. This was done by applying a separate linear regression per vertebral level and brainstem structure using the baseline data from the healthy control group. Predicted volumes were calculated for all subjects, and z‐scores were computed by subtracting the predicted volume from the actual volume and dividing this by the SD of the residuals of the linear regression model. This approach ensures that age effects are removed and that repeated measurements from the same subject do not artificially reduce the residual variance.

To uniformly quantify sensitivity to change for the spinal cord and brainstem structures, individual change scores were calculated as the difference between the 1‐year follow‐up and baseline measurements. The SRM was then calculated by dividing the mean of these individual changes in CSA and brainstem structure volume by their standard deviation. A 95% CI was computed for each SRM value.

Relationships between structural and clinical progression were assessed in symptomatic participants. Associations were examined between baseline CSA and baseline SARA scores, and between percent CSA change (% change) at C2 and change in SARA score (ΔSARA). Next, we determined the predictive value of the expanded CAG repeat length and the s‐factor $\left(\frac{\text{Expanded repeat length}-\text{Normal repeat length}}{\text{Normal repeat length}}\times \left(\text{current}\;\mathrm{age}-\mathrm{age}\;\mathrm{at}\;\text{disease onset}\right)\times 10\right)$ to take disease burden into account for subsequent atrophy in both the preataxic and symptomatic group, adjusting for age and sex. To further investigate these associations, CSA was compared between SCA1 expansion carriers exhibiting spasticity (yes/no = 25/34) or sensory symptoms (yes/no = 26/34) and those without these symptoms, based on INAS sub‐scored. Linear models adjusted for age, sex, and disease duration were used, with Bonferroni‐Holm correction for multiple testing. Differences in disease severity and duration between subgroups were assessed using Welch two‐sample *t* tests. Additional sample size and power analyses simulated 1‐year clinical trials to estimate the number of participants required to detect volumetric changes in the pons and C2, as well as SARA progression, across a range of assumed effect sizes. These analyses assessed the feasibility and sensitivity of these biomarkers in trial‐relevant settings.

All statistical analyses were conducted using R version [4.5.1] and RStudio version 2025.5.1.513.^34^

## Results

### Study Cohort

In this study, 40 controls, 16 preataxic, and 58 symptomatic carriers were included (Table 1, Fig. S2). At baseline, the number of participants with available data varied across groups and cervical spinal cord levels (Table S1).

**TABLE 1:** Demographics with mean characteristics at baseline of all participants

	Control	Preataxic SCA1	Symptomatic SCA1
**No. of participants**			
Baseline	40	16	58
1‐yr follow‐up	23	9	41
**Mean age [range]**			
Baseline	41 [19–74]	32 [18–50]	48 [18–74]
1‐yr follow‐up	46 [20–75]	38 [21–51]	52 [31–75]
**Gender female/male**			
Baseline	27/13	6/10	27/31
1‐yr follow‐up	13/10	3/6	20/21
**Expanded CAG‐repeat [range]**			
Baseline	NA	45.8 [40.0–50.0]	44.9 [38.0–57.0]
1‐yr follow‐up	NA	46.2 [43.0–50.0]	45.1 [38.0–57.0]
**Age at onset (yr)**			
Baseline	NA	NA	42.6 ± 12.1
1‐yr follow‐up	NA	NA	43.6 ± 11.2
**Disease duration (yr)**			
Baseline	NA	−6.3 ± 10.0*	6.6 ± 5.6
1‐yr follow‐up	NA	−5.5 ± 2.5*	7.4 ± 5.5
**Mean SARA score ± SD**			
Baseline	0.4 ± 0.6	0.9 ± 1.1	11.2 ± 6.3
1‐yr follow‐up	0.0 ± 0.2	1.2 ± 1.1	12.7 ± 7.4
**Mean follow‐up time ± SD**	1.03 ± 0.11	0.98 ± 0.07	1.03 ± 0.08

* Predicted time to onset for preataxic participants.

Abbreviations: SCA1, spinocerebellar ataxia type 1; SARA, Scale for the Assessment and Rating of Ataxia; SD, standard deviation.

### Cross‐Sectional Analysis

**FIG. 1:**
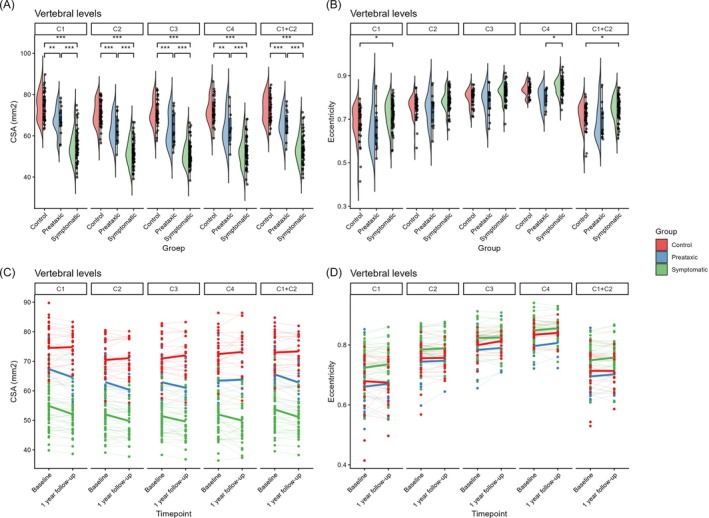
Cross‐sectional area and eccentricity at vertebral levels C1, C2, C3, C4, and C1 and C2 combined at (**A**,**B**) baseline and (**C**,**D**) 1‐year follow‐up with subject‐specific trajectories. [Color figure can be viewed at wileyonlinelibrary.com]

Separate one‐way analysis of variances (ANOVAs) were conducted for each cervical spinal cord level to assess group differences. At baseline, there was a significant main effect of group on cervical spinal cord CSA (Fig. 1A) and eccentricity (Fig. 1B) at levels C1 to C4 (Table S2) (all *P* < 0.001). Post‐hoc pairwise comparisons revealed that symptomatic patients had significantly smaller CSA compared to controls (all *P* < 0.001) and preataxic carriers (all *P* < 0.001). In addition, preataxic carriers showed significantly reduced CSA compared to controls at levels C1 to C4 (Table S3) C1–C4 and C1 + C2 *P* ≤ 0.001). In contrast, post‐hoc test for baseline eccentricity comparison indicated only a significantly higher eccentricity in the symptomatic group compared with the control group at C1 (β = −0.055, *P* = 0.020) and C1 + C2 (β = −0.044, *P* = 0.034), and for level C4 there was a significant difference between preataxic participants and symptomatic patients (β = −0.049, *P* = 0.010).

### Longitudinal Analyses

Longitudinal analyses indicated that CSA loss was not uniform along the cervical cord, but was particularly evident at the upper cervical spinal cord levels (Fig. 1C,D). At C1 (*P* = 0.005), C2 (*P* = 0.002), and C1–C2 (*P* = 0.001), significant group differences in 1‐year atrophy rates were observed (Table S4). Post‐hoc analyses revealed that preataxic carriers showed a significant decline in CSA at C1 (β = 2.635, *P* < 0.001), C2 (β = 2.067, *P* = 0.003), and C1–C2 (β = 2.442, *P* < 0.001), whereas symptomatic patients declined significantly at C1 (β = 1.029, *P* = 0.003), C2 (β = 0.828, *P* = 0.013), and C1–C2 (β = 0.955, *P* = 0.001). Controls did not show a significant CSA decline at any level (Table S5). For eccentricity, the C1 (*P* = 0.006), C2 (*P* = 0.011), and C1–C2 (*P* = 0.001) levels showed a significant interaction between group and time (Table S4). Post‐hoc tests showed a significant difference over time for the preataxic carriers at C2 (β = −0.016, *P* = 0.016) and C1–C2 level (β = −0.017, *P* = 0.020), and the control group showed a significant difference over time at C1–C2 level (β = 0.009, *P* = 0.046). Symptomatic patients did not show a significant difference over time (Table S5).

### Age‐Adjusted Temporal Dynamics

**FIG. 2:**
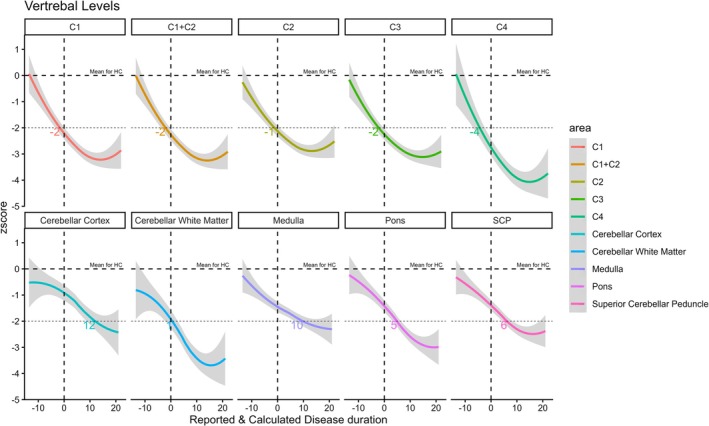
Z‐score of spinal cord cross‐sectional area (CSA) (top row) and brainstem and cerebellar volumes (bottom row) versus disease duration (symptomatic reported, preataxic calculated). Vertical dashed line: estimated age of onset (0); long‐dashed horizontal line: healthy control mean; short dashed line with numbers: estimated disease duration (years) at *z* = −2. [Color figure can be viewed at wileyonlinelibrary.com]

Comparisons between spinal cord and brainstem measures suggested that spinal cord atrophy progresses more rapidly and precedes pontine atrophy during the disease course. When normalized z‐scores of CSA, brainstem, and cerebellar measures were plotted against disease duration, preataxic carriers already exhibited a stronger decline in spinal cord z‐score (Fig. 2). CSA at all vertebral levels reached a z‐score of −2 approximately 1–4 years before symptom onset, indicating CSA changes clearly precede clinical manifestation. In contrast, pontine z‐scores reached −2 approximately 5 years after onset, changes in other brainstem and cerebellar regions were more gradual and less pronounced.

### Stage‐Dependent Sensitivity to Changes

**FIG. 3:**
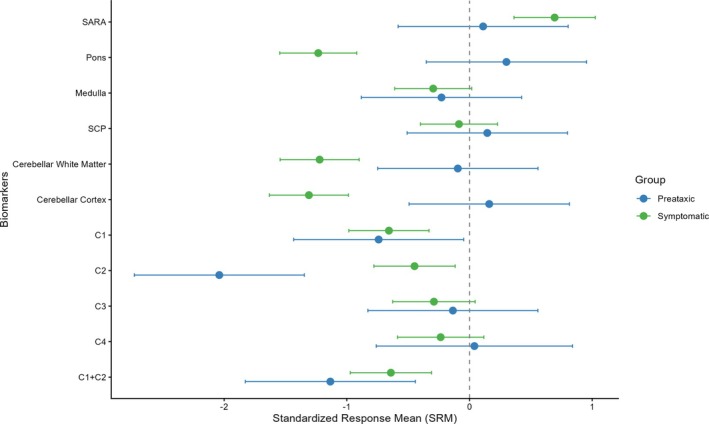
The standardized response mean (SRM) for cross‐sectional area (CSA) at all vertebral levels, for volume of all brainstem structures, and for the total Scale for the Assessment and Rating of Ataxia (SARA) score comparing the preataxic and symptomatic spinocerebellar ataxia type 1 (SCA1) group. [Color figure can be viewed at wileyonlinelibrary.com]

When universally comparing sensitivity to change between cervical CSAs, cerebellar, and brainstem measures, the C2 CSA showed the highest responsiveness within the subgroup of preataxic subjects (SRM = −2.0), followed by the combination of C1–C2 CSA (SRM = −1.1). In symptomatic patients, SRMs for C2 (SRM = −0.4) and C1–C2 (SRM = −0.6) were lower and were outperformed by pontine volume (SRM = −1.2) in this subgroup (Fig. 3).

### Clinical Associations

**FIG. 4:**
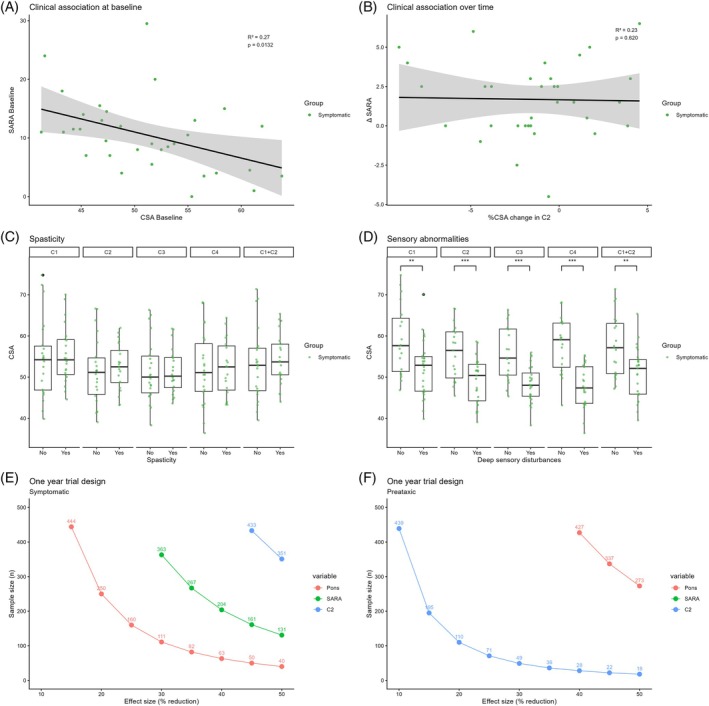
Clinical associations (**A**) between baseline cross‐sectional area (CSA) at C2 and Scale for the Assessment and Rating of Ataxia (SARA) score in symptomatic patients and (**B**) %change in CSA at C2 and ΔSARA in symptomatic participants. CSA stratified by the non‐ataxia clinical features (**C**) spasticity (yes/no) and (**D**) sensory abnormalities (yes/no). Trial design: sample size versus effect size for a one‐arm trial in (**E**) symptomatic and (**F**) preataxic participants. [Color figure can be viewed at wileyonlinelibrary.com]

To examine the possible clinical correlates of spinal cord atrophy in SCA1 associations between structural measures and disease severity were assessed in symptomatic participants. CSA at C2 was significantly associated with baseline SARA score (Fig. 4A) (β = −0.451, *P* = 0.013), whereas 1‐year C2 volume change was not associated with change in SARA. (Fig. 4B) (β = −0.057, *P* = 0.620). Across preataxic and symptomatic expansion carriers, neither S‐factor nor CAG repeat length was associated with CSA change, and CSA atrophy did not correlate with spasticity at any cervical level (Fig. 4C). In contrast, CSA was significantly reduced in individuals with sensory abnormalities (Fig. 4D) across multiple cervical levels (C1; β = −6.841, *P* = 0.010, C2; β = −7.323, *P* = 0.001, C3; β = −7.476, *P* = 0.001, C4; β = −10.546, *P* < 0.001, C1 + C2; β = −6.815, *P* = 0.006) after adjustment for age, sex, and disease duration. Sensory disturbance was associated with disease duration at the levels C2, C3, and C4, and patients with sensory disturbances had a significantly longer disease duration compared to those without (7.45 vs. 0.02 years; *P* < 0.001).

Finally, we calculated the required sample sizes for interventional trials based on C2 CSA, pontine volume, or SARA score as endpoints, with separate analyses for symptomatic patients (Fig. 4E) and preataxic carriers (Fig. 4F). Our results indicate that an interventional trial in preataxic carriers focusing on reduction of rate of atrophy would require substantially less patients if CSA at C2 level would be the endpoint, compared to pontine volume. In symptomatic patients, C2 level was less sensitive to change, and targeting reduction in rate of pontine atrophy or reduction of SARA progression seemed more effective to optimize required trial sample sizes.

## Discussion

This imaging study demonstrates that cervical spinal cord atrophy, is a clear pathological hallmark across all SCA1 disease stages, and that measurable spinal cord changes are already present at the preataxic stage. These findings are compatible with, but do not robustly establish, a caudal to rostral progression of degenerative changes. Cervical spinal cord atrophy was most pronounced at the upper vertebral levels. In comparison to other imaging markers, C2 atrophy showed most sensitivity to change at the preclinical stage, whereas for the symptomatic disease phase this was true for pontine volume. Although in symptomatic expansion carriers, pontine volume loss is known to be more associated with worsening of clinical score,^3^ C2 atrophy was associated with baseline SARA score, but not with SARA progression.

Overall, our results indicate that cervical spinal cord imaging, in particular of the higher cervical levels, can serve as a biomarker for radiological disease progression before clinical manifestation and might have potential as a sensitive outcome measure in interventional trials. Specifically, we found that using C2 CSA in clinical trials targeting the preataxic stage would require substantially fewer participants to detect an effect on the rate of atrophy compared with pontine volume, highlighting the particular sensitivity of upper cervical cord measures to early disease‐relevant changes. In contrast, in symptomatic patients, C2 CSA was less sensitive to change over time, and endpoints such as pontine atrophy or SARA score progression have been shown to be more efficient for optimizing trial sample sizes.^35^

These results add to the evidence that spinal cord degeneration is a pathological process in SCA1, alongside or more likely before the well‐documented cerebellar and brainstem atrophy.^3^ Postmortem neuropathological studies in SCA1 have demonstrated neurodegeneration extending beyond the cerebellum to involve the brainstem and spinal cord, including degeneration of long tracts and brainstem nuclei, particularly in advanced disease stages.^9^, ^36^, ^37^ Neuroimaging studies have largely mirrored these findings, with prominent cerebellar, pons, and brainstem atrophy reported in symptomatic SCA1 subjects and associations with clinical severity.^7^, ^35^, ^38^, ^39^ Recent studies in SCA1 and SCA3 demonstrated that pons volume is one of the most sensitive endpoints at both the preataxic and early ataxic stages, outperforming clinical scores such as SARA, and supporting the feasibility of preventive trials using infratentorial MRI measures in polyglutamine SCAs.^14^, ^40^, ^41^ However, the temporal and spatial progression of pathology remains incompletely characterized, particularly in SCA1.

Specifically, atrophy of higher cervical levels may emerge before detectable cerebellar and brainstem involvement.^6^, ^8^ Similar atrophy patterns were reported in related ataxias such as SCA2 and SCA3, where CSA loss correlated with disease severity and duration.^6^ However, there seems to be SCA subtype‐specific changes, for instance, eccentricity changes appear more specific to certain SCAs such as SCA2, highlighting disease‐specific spinal cord morphometric patterns.^6^, ^42^ Our findings indicate that eccentricity may be a less robust and sensitive marker of spinal cord degeneration in SCA1. Eccentricity changes did not consistently distinguish the three groups, and changes were observed only at the highest cervical levels (C1 and C1–C2). Interestingly, a significant change in eccentricity over time was also observed in the control group, which was unexpected. These observations suggest that eccentricity might be influenced by local anatomical variability or measurement sensitivity, limiting its utility as a stage or monitoring.

Spinal cord atrophy might be the result of both axonal and myelin pathology as previously shown in recent imaging studies using diffusion metrics to assess white matter alterations.^43^ These findings could point to the involvement of oligodendrocyte dysfunction and altered myelin maintenance as contributing mechanisms in SCA1. Such insights reinforce a broader disease model in which spinal cord pathology, both axonal and glial, may provide additional therapeutic targets for intervention.^14^, ^44^ Moreover, integrating spinal cord metrics into multimodal biomarker panels alongside brainstem and cerebellar imaging could substantially improve early disease detection, patient stratification, and the evaluation of treatment efficacy in future clinical trials.

We did not find a direct association between spinal cord CSA loss and SARA progression. This likely reflects the fact that C2 atrophy is already pronounced in preataxic carriers, in whom there is no clinical manifestation of ataxia per definition. In symptomatic participants, ataxia severity and progression is likely driven more by cerebellar and brainstem involvement, which may explain the lack of a longitudinal association with C2 atrophy. The observed baseline relationship would then reflect ongoing atrophy during the symptomatic disease phase without an independent contribution to ataxia severity. We did find evidence to connect CSA decrease to deep sensory disturbance that could be partly attributed to spinal cord dysfunction and more specifically to involvement of the dorsal columns, whereas we found no such association for spasticity. This association remained significant after controlling for disease duration, suggesting that spinal cord degeneration contributes to sensory deficits independently of general disease progression. Lack of this association in our study might be partly related to the binary rather than more granular design of the INAS.^45^ An additional limitation is that no electromyography data were available to examine peripheral neuropathy as substrate for the sensory deficits. Further studies are needed to determine if spinal cord atrophy can be robustly linked to specific, long term clinically meaningful and relevant changes in SCA1 patients (eg, in digital motor measures capturing gait changes in SCA1).

This study has several limitations. No dedicated spinal cord coil was used, however, imaging of the upper cervical cord with standard head/neck coils is feasible. Although off‐isocenter positioning may introduce residual geometric distortion and B0 inhomogeneities, these effects were likely limited at C1–C4. Gray/white matter segmentation would require proton density‐ or intermediate‐weighted sequences,^46^, ^47^ which were not included in our protocol, restricting insights into affected spinal pathways and demyelination.^48^ These technical constraints may have underestimated regional pathological heterogeneity and precluded pathway‐specific analyses crucial for mechanistic understanding. Future studies could, therefore, incorporate more fine‐grained subregional segmentation approaches to improve neuroanatomical precision and facilitate pathway‐specific mechanistic insights. Combined exploration of oligodendrocyte pathology and myelin integrity through advanced imaging and molecular markers may reveal the cellular mechanisms underlying spinal cord degeneration in SCA1. Additionally, although our study was built on one of the largest aggregated SCA1 datasets so far, the number of preataxic carriers was relatively low and future studies should explicitly secure sufficient coverage of all SCA1 disease stages.

In conclusion, our results show that cervical spinal cord atrophy, particularly at upper level C2, is an early and progressive imaging feature of SCA1 detectable in preataxic stage. This spinal cord degeneration may precede or occur in parallel with cerebellar and brainstem pathology and represents a promising imaging biomarker for preclinical and early symptomatic disease phases. However, formal group‐level analyses comparing brainstem structures across disease stages are required to directly test the temporal sequence of these changes. To fully establish cervical spinal cord CSA as a reliable biomarker in SCA1, future studies should investigate its predictive value for disease conversion and clinically meaningful decline. Such work could enable early detection, improve prognostic stratification, and support the use of CSA as a surrogate endpoint in interventional (preventive) trials.

## Author Roles

(1) Research project: A. Conception, B. Organization, C. Execution; (2) Statistical Analysis: A. Design, B. Execution, C. Review and Critique; (3) Manuscript: A. Writing of the First Draft, B. Review and Critique.

C.R.: 1B, 1C, 2A, 2B, 3A

T.v.P.: 1B, 1C, 2C, 3B

K.K.: 1B, 1C, 2C, 3B

J.v.A.: 1C, 3B

C.M.: 1C, 3B

L.S.: 1C, 3B

A.N.: 1C, 3B

M.G.: 3B

G.C.: 1C, 3B

A.D.: 3B

T.S.: 1A, 3B

B.v.d.W.: 1A, 2C, 3B

## Financial Disclosures and Conflicts of Interest:

B.v.d.W. has received consulting fees from Biogen, Vico Therapeutics, and Biohaven Pharmaceuticals and financial support for participating in the advisory board of Vico Therapeutics. B.v.d.W. receives financial support from employment at the Radboud University Medical Center. B.v.d.W. has received a reviewer fee for Gossweiler Foundation and royalties from BSL/Springer Nature and reports grants from Hersenstichting, ZonMw, Dutch Research Council, and Christina Foundation. L.S. received consulting fees from Novartis Farma S.p.A., C.M. received consulting fees from Biogen and Novartis. C.R., T.v.P., K.K., J.v.A., A.N., M.G., G.C., A.D., K.D., and T.S. have no disclosures to report.

## Supporting information


**Figure S1.** Schematic overview of the SCT pipeline. The first four panels show individual examples from the study participant. including resampled. normalized. segmented. and labeled MPRAGE images. The last panel show example drawings illustrating cross‐sectional area (mm^2^) and eccentricity (focal distance/major axis). adapted from [https://spinalcordtoolbox.com]. Values are calculated for each vertebral level. with numbers representing data from a representative participant.
**Figure S2.** Site distribution of the groups.
**Table S1.** Count and averages CSA and Eccentricity.
**Table S2.** Baseline ANOVA type III group results reported as numerator degrees of freedom (NumDF) and denominator degrees of freedom (DenDF), the *F*‐value and *p*‐value.
**Table S3.** Pair‐wise posthoc results comparing group, (Pre = preataxic and Symp = symptomatic).
**Table S4.** Longitudinal ANOVA Type III group × session interaction results of the reported as numerator degrees of freedom (NumDF) and denominator degrees of freedom (DenDF) the *F*‐value and *p*‐value.
**Table S5.** Post‐hoc pair wise comparison of baseline and 1‐year follow up CSA and Eccentricity measures per group (Pre = Preataxic and Symp = Symptomatic) and area.

## Data Availability

Requests for data sharing can be directed to the corresponding author and will be reviewed by the senior author.
