# Supplementary material for: Early and Progressive Spinal Cord Atrophy in Spinocerebellar Ataxia Type 1
Source: Mov Disord. 2026 Apr 9;41(7):1836–46. doi: 10.1002/mds.70294 (PMC13387905; doi:10.1002/mds.70294)
Supplement: Supplementary file 1 — Figure S1. Schematic overview of the SCT pipeline. The first four panels show individual examples from the study participant. including resampled. normalized. segmented. and labeled MPRAGE images. The last panel show example drawings illustrating cross‐sectional area (mm2) and eccentricity (focal distance/major axis). adapted from [https://spinalcordtoolbox.com]. Values are calculated for each vertebral level. with numbers representing data from a representative participant. Figure S2. Site distribution of the groups. Table S1. Count and averages CSA and Eccentricity. Table S2. Baseline ANOVA type III group results reported as numerator degrees of freedom (NumDF) and denominator degrees of freedom (DenDF), the F‐value and p‐value. Table S3. Pair‐wise posthoc results comparing group, (Pre = preataxic and Symp = symptomatic). Table S4. Longitudinal ANOVA Type III group × session interaction results of the reported as numerator degrees of freedom (NumDF) and denominator degrees of freedom (DenDF) the F‐value and p‐value. Table S5. Post‐hoc pair wise comparison of baseline and 1‐year follow up CSA and Eccentricity measures per group (Pre = Preataxic and Symp = Symptomatic) and area. [file MDS-41-1836-s001.docx]

# **Supplementary**


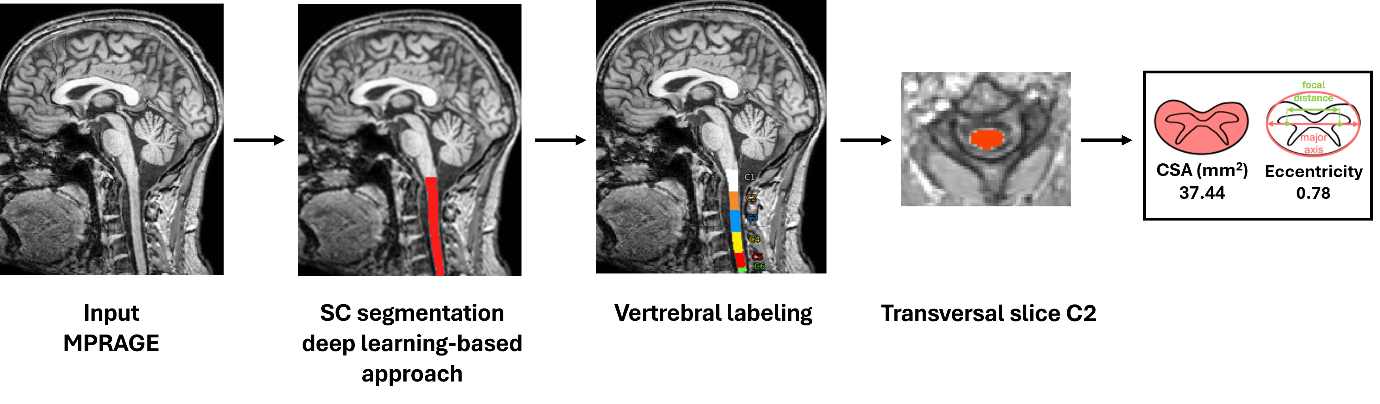


***Figure S1****. Schematic overview of the SCT pipeline. The first four panels show individual examples from the study participant, including resampled, segmented, and labeled MPRAGE images. The last panel show example drawings illustrating cross-sectional area (mm²) and eccentricity (focal distance / major axis), adapted from [*[*https://spinalcordtoolbox.com*](https://spinalcordtoolbox.com/)*]. Values are calculated for each vertebral level, with numbers representing data from a representative participant.*

**Figure S2.** Site distribution of the groups.


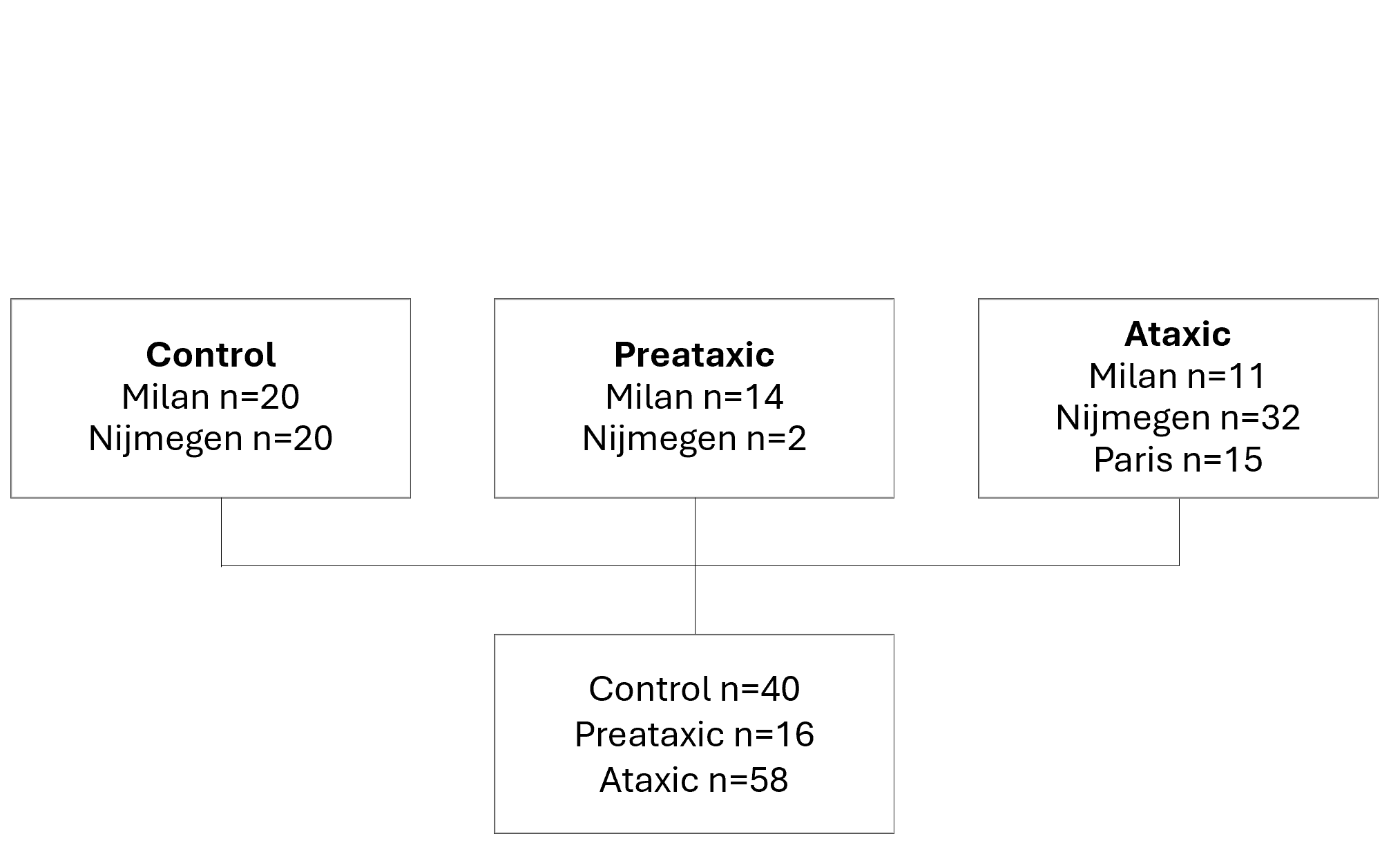


**Table S1**. Count and averages CSA and Eccentricity.

|  | **Vertebral level** | **Control** | | **Preataxic SCA1** | | **Symptomatic SCA1** | |
| --- | --- | --- | --- | --- | --- | --- | --- |
|  |  | **ses-01** | **ses-02** | **ses-01** | **ses-02** | **ses-01** | **ses-02** |
| Count | C1 | 38 | 21 | 15 | 8 | 55 | 38 |
|  | C2 | 38 | 21 | 15 | 8 | 54 | 37 |
|  | C3 | 36 | 19 | 15 | 8 | 53 | 36 |
|  | C4 | 32 | 17 | 10 | 7 | 51 | 33 |
|  | C1+C2 | 37 | 20 | 15 | 8 | 54 | 37 |
| Average CSA (mm2) | C1 | 75.22 | 74.80 | 67.38 | 64.53 | 54.88 | 52.08 |
|  | C2 | 71.24 | 71.05 | 62.94 | 60.38 | 52.02 | 49.78 |
|  | C3 | 71.73 | 72.00 | 62.90 | 61.05 | 51.44 | 49.57 |
|  | C4 | 72.45 | 73.15 | 63.44 | 63.77 | 51.98 | 49.92 |
|  | C1+C2 | 73.72 | 73.33 | 65.56 | 62.80 | 53.68 | 51.11 |
| Average Eccentricity | C1 | 0.68 | 0.67 | 0.66 | 0.67 | 0.72 | 0.74 |
|  | C2 | 0.75 | 0.76 | 0.74 | 0.75 | 0.78 | 0.79 |
|  | C3 | 0.80 | 0.81 | 0.78 | 0.79 | 0.82 | 0.83 |
|  | C4 | 0.83 | 0.84 | 0.80 | 0.81 | 0.85 | 0.86 |
|  | C1+C2 | 0.71 | 0.71 | 0.70 | 0.70 | 0.75 | 0.76 |

**Table S2**. Baseline ANOVA type III group results are reported as numerator degrees of freedom (NumDF) and denominator degrees of freedom (DenDF), F-value and p-value.

|  | **Level** | **Num df** | **Den df** | **F-value** | **p-value** |
| --- | --- | --- | --- | --- | --- |
| CSA | C1 | 2 | 98.199 | 81.078 | <0.001 |
|  | C2 | 2 | 93.662 | 84.922 | <0.001 |
|  | C3 | 2 | 80.706 | 80.871 | <0.001 |
|  | C4 | 2 | 82.453 | 80.720 | <0.001 |
|  | C1+C2 | 2 | 95.422 | 85.088 | <0.001 |
| Eccentricity | C1 | 2 | 100.079 | 5.920 | 0.004 |
|  | C2 | 2 | 90.183 | 5.335 | 0.006 |
|  | C3 | 2 | 62.886 | 4.712 | 0.012 |
|  | C4 | 2 | 68.032 | 6.620 | 0.002 |
|  | C1+C2 | 2 | 97.314 | 5.757 | 0.004 |

**Table S3**. Post hoc pairwise comparisons of the control, preataxic (Pre), and symptomatic (Symp) groups.

|  | **Level** | **contrast** | **β** | **SE** | **df** | **T-ratio** | **Lower CL** | **Upper CL** | **p-value** | **Adj. p-value** |
| --- | --- | --- | --- | --- | --- | --- | --- | --- | --- | --- |
| CSA | C1 | Control - Pre | 8.948 | 2.391 | 101.905 | 3.743 | 3.262 | 14.635 | 0.001 | 0.001 |
|  |  | Control - Symp | 22.383 | 1.827 | 100.129 | 12.251 | 18.036 | 26.729 | <0.001 | <0.001 |
|  |  | Pre - Symp | 13.435 | 2.578 | 101.091 | 5.211 | 7.302 | 19.567 | <0.001 | <0.001 |
|  | C2 | Control - Pre | 9.726 | 2.193 | 101.140 | 4.435 | 4.510 | 14.943 | <0.001 | <0.001 |
|  |  | Control - Symp | 20.929 | 1.688 | 95.334 | 12.402 | 16.911 | 24.947 | <0.001 | <0.001 |
|  |  | Pre - Symp | 11.203 | 2.380 | 97.291 | 4.707 | 5.538 | 16.867 | <0.001 | <0.001 |
|  | C3 | Control - Pre | 10.082 | 2.265 | 98.605 | 4.450 | 4.691 | 15.473 | <0.001 | <0.001 |
|  |  | Control - Symp | 21.206 | 1.786 | 80.839 | 11.871 | 16.941 | 25.472 | <0.001 | <0.001 |
|  |  | Pre - Symp | 11.124 | 2.483 | 83.810 | 4.480 | 5.199 | 17.049 | <0.001 | <0.001 |
|  | C4 | Control - Pre | 10.001 | 2.551 | 87.087 | 3.920 | 3.918 | 16.084 | 0.001 | 0.001 |
|  |  | Control - Symp | 21.805 | 1.808 | 82.680 | 12.063 | 17.491 | 26.119 | <0.001 | <0.001 |
|  |  | Pre - Symp | 11.804 | 2.684 | 86.182 | 4.397 | 5.402 | 18.206 | <0.001 | <0.001 |
|  | C1+C2 | Control - Pre | 9.231 | 2.261 | 99.921 | 4.082 | 3.851 | 14.611 | <0.001 | <0.001 |
|  |  | Control - Symp | 22.056 | 1.764 | 97.208 | 12.503 | 17.857 | 26.254 | <0.001 | <0.001 |
|  |  | Pre - Symp | 12.825 | 2.463 | 98.505 | 5.206 | 6.963 | 18.686 | <0.001 | <0.001 |
| Eccentricity | C1 | Control - Pre | 0.005 | 0.023 | 100.734 | 0.217 | -0.051 | 0.061 | 0.974 | 0.974 |
|  |  | Control - Symp | -0.055 | 0.018 | 101.216 | -3.109 | -0.098 | -0.013 | 0.007 | 0.020 |
|  |  | Pre - Symp | -0.060 | 0.025 | 101.531 | -2.410 | -0.120 | -0.001 | 0.046 | 0.093 |
|  | C2 | Control - Pre | 0.010 | 0.017 | 100.546 | 0.578 | -0.031 | 0.051 | 0.832 | 0.832 |
|  |  | Control - Symp | -0.036 | 0.013 | 89.330 | -2.699 | -0.067 | -0.004 | 0.022 | 0.067 |
|  |  | Pre - Symp | -0.046 | 0.019 | 91.303 | -2.447 | -0.090 | -0.001 | 0.043 | 0.085 |
|  | C3 | Control - Pre | 0.014 | 0.015 | 94.998 | 0.948 | -0.021 | 0.049 | 0.612 | 0.612 |
|  |  | Control - Symp | -0.025 | 0.011 | 60.945 | -2.164 | -0.052 | 0.003 | 0.086 | 0.172 |
|  |  | Pre - Symp | -0.039 | 0.016 | 60.354 | -2.436 | -0.077 | -0.001 | 0.046 | 0.139 |
|  | C4 | Control - Pre | 0.036 | 0.014 | 84.982 | 2.544 | 0.002 | 0.070 | 0.034 | 0.068 |
|  |  | Control - Symp | -0.013 | 0.010 | 63.417 | -1.350 | -0.037 | 0.010 | 0.373 | 0.373 |
|  |  | Pre - Symp | -0.049 | 0.015 | 70.349 | -3.369 | -0.085 | -0.014 | 0.003 | 0.010 |
|  | C1+C2 | Control - Pre | 0.010 | 0.019 | 98.816 | 0.497 | -0.036 | 0.056 | 0.873 | 0.873 |
|  |  | Control - Symp | -0.044 | 0.015 | 98.297 | -2.940 | -0.080 | -0.008 | 0.011 | 0.034 |
|  |  | Pre - Symp | -0.054 | 0.021 | 98.909 | -2.569 | -0.104 | -0.004 | 0.031 | 0.062 |

**Table S4**. Longitudinal ANOVA Type III group × session interaction results are reported as numerator degrees of freedom (NumDF), denominator degrees of freedom (DenDF), F-values, and p-values.

|  | **Level** | **NumDf** | **DenDf** | **F-value** | **p-value** |
| --- | --- | --- | --- | --- | --- |
| CSA | C1 | 2 | 61.443 | 5.779 | 0.005 |
|  | C2 | 2 | 60.452 | 7.112 | 0.002 |
|  | C3 | 2 | 57.684 | 1.734 | 0.186 |
|  | C4 | 2 | 53.261 | 1.051 | 0.357 |
|  | C1+C2 | 2 | 59.312 | 7.933 | 0.001 |
| Eccentricity | C1 | 2 | 60.750 | 5.519 | 0.006 |
|  | C2 | 2 | 59.621 | 4.843 | 0.011 |
|  | C3 | 2 | 54.737 | 2.607 | 0.083 |
|  | C4 | 2 | 50.192 | 2.560 | 0.087 |
|  | C1+C2 | 2 | 58.546 | 7.460 | 0.001 |

**Table S5** Post hoc pairwise comparisons of baseline and 1-year follow-up CSA and eccentricity measures per group (Pre = preataxic; Symp = symptomatic) and area.

|  | **Level** | **group** | **β** | **SE** | **df** | **T-ratio** | **Lower CL** | **Upper CL** | **p-value** | **Adj. p-value** |
| --- | --- | --- | --- | --- | --- | --- | --- | --- | --- | --- |
| CSA | C1 | Control | 0.051 | 0.416 | 64.645 | 0.122 | -0.780 | 0.882 | 0.903 | 0.903 |
|  |  | Pre | 2.635 | 0.654 | 62.941 | 4.030 | 1.328 | 3.941 | <0.001 | <0.001 |
|  |  | Symp | 1.029 | 0.313 | 66.192 | 3.286 | 0.404 | 1.655 | 0.002 | 0.003 |
|  | C2 | Control | -0.452 | 0.385 | 63.704 | -1.174 | -1.222 | 0.318 | 0.245 | 0.245 |
|  |  | Pre | 2.067 | 0.605 | 61.925 | 3.416 | 0.857 | 3.277 | 0.001 | 0.003 |
|  |  | Symp | 0.828 | 0.294 | 65.283 | 2.814 | 0.241 | 1.416 | 0.006 | 0.013 |
|  | C1+C2 | Control | 0.069 | 0.333 | 63.578 | 0.208 | -0.596 | 0.734 | 0.836 | 0.836 |
|  |  | Pre | 2.442 | 0.508 | 61.141 | 4.808 | 1.427 | 3.458 | <0.001 | <0.001 |
|  |  | Symp | 0.955 | 0.249 | 66.431 | 3.839 | 0.458 | 1.452 | <0.001 | 0.001 |
| Eccentricity | C1 | Control | 0.014 | 0.006 | 63.117 | 2.368 | 0.002 | 0.025 | 0.021 | 0.063 |
|  |  | Pre | -0.017 | 0.009 | 62.168 | -1.892 | -0.035 | 0.001 | 0.063 | 0.126 |
|  |  | Symp | -0.006 | 0.004 | 63.531 | -1.357 | -0.014 | 0.003 | 0.180 | 0.180 |
|  | C2 | Control | 0.005 | 0.004 | 62.077 | 1.232 | -0.003 | 0.012 | 0.223 | 0.445 |
|  |  | Pre | -0.016 | 0.006 | 60.952 | -2.886 | -0.028 | -0.005 | 0.005 | 0.016 |
|  |  | Symp | -0.001 | 0.003 | 62.943 | -0.424 | -0.007 | 0.004 | 0.673 | 0.673 |
|  | C1+C2 | Control | 0.009 | 0.004 | 61.226 | 2.336 | 0.001 | 0.017 | 0.023 | 0.046 |
|  |  | Pre | -0.017 | 0.006 | 59.888 | -2.809 | -0.029 | -0.005 | 0.007 | 0.020 |
|  |  | Symp | -0.004 | 0.003 | 62.656 | -1.400 | -0.010 | 0.002 | 0.166 | 0.166 |
